# Mobile radiography services in nursing homes: a systematic review of residents’ and societal outcomes

**DOI:** 10.1186/s12913-017-2173-8

**Published:** 2017-03-23

**Authors:** Elin Kjelle, Kristin Bakke Lysdahl

**Affiliations:** 1 Department of Optometry, Radiography and Lighting Design, Faculty of Health and Social Sciences, University College of Southeast Norway, Postboks 235, 3603 Kongsberg, Norway; 2 Institute of radiography and dental technology, Department of Life Sciences and Health, Faculty of health sciences, Oslo and Akershus University College of Applied Sciences, Postboks 4, St. Olavs plass, 0130 Oslo, Norway

**Keywords:** Mobile radiography service, Nursing homes, Socio-economics, X-ray, Telemedicine

## Abstract

**Background:**

Demographic changes are leading to an ageing population in Europe, and predict an increase in the number of nursing home residents over the next 30 years. Nursing home residents need specialised healthcare services such as radiology due to both chronic and acute illnesses. Mobile radiography, x-ray examinations performed in the nursing homes, may be a good way of providing services to this population. The aim of this systematic review was to identify the outcomes of mobile radiography services for nursing home residents and society.

**Methods:**

A systematic review based on searches in the Medline, Cochrane, PubMed, Embase and Svemed + databases was performed. Titles and abstracts were screened according to a predefined set of inclusion criteria: empirical studies in the geriatric population, and reports of mobile radiography services in a clinical setting. All publications were quality appraised using MMAT or CASP appraisal tools. Data were extracted using a summary table and results were narratively synthesised.

**Results:**

Ten publications were included. Three overarching outcomes were identified: 1) reduced number of hospitalisations and outpatient examinations or treatments, 2) reduced number of transfers between nursing homes and hospitals and 3) increased access to x-ray examinations. These outcomes were interlinked with the more specific outcomes for residents and society reported in the literature. For residents there was a reduction in burdensome transfers and waiting time and adequate treatment and care increased. For society, released resources could be used more efficiently, and overall costs were reduced substantially.

**Conclusions:**

This review indicates that mobile radiography services for nursing home residents in the western world are of comparable quality to hospital-based examinations and have clear potential benefits. Mobile radiography reduced transfers to and from hospital, increased the number of examinations carried out and facilitated timely diagnosis and access to treatments. Further research is needed to formally evaluate potential improvements in care quality and cost-effectiveness.

**Electronic supplementary material:**

The online version of this article (doi:10.1186/s12913-017-2173-8) contains supplementary material, which is available to authorized users.

## Background

Increased interest in demographic changes in our society leading to an ageing population highlights the need for healthcare services to be more effective maintaining high quality standards [1]. An increase in the number of persons living in nursing homes is expected over the next 30 years [1]. Today, nursing home residents are living with several chronic illnesses and up to 80% have dementia [2–4]. In addition, there is a high incidence of acute illnesses such as infections, cardiovascular incidents and injury due to falls [3, 4]. Both chronic illnesses and acute illnesses increase the need for specialist healthcare services for these residents compared to the rest of the population [3]. According to Graverholt and Riise [4] almost 45% of admissions to hospital from nursing homes are related to falls, respiratory infections and diseases of the digestive system [4]. For these indications, conventional x-ray examinations such as chest, musculoskeletal and abdominal images are important diagnostic tests [5, 6]. Today, nursing home residents often require transfer to a hospital or an emergency room (ER) to attend radiological services. The sudden change in environment for the nursing home residents, and especially persons suffering from dementia, can affect the person’s orientation and sense of security. Transportation and new surroundings such as the x-ray department in a hospital may lead to increased anxiety or disorientation [7]. Inadvertently transfer to hospital may do more harm than good for a nursing home resident, thus hospitalisation should be avoided [2, 5]. In addition, the transfer may affect cost and acceptability of radiological services [8].

It is possible to perform conventional x-ray examinations in nursing homes as a telemedicine service [8, 9]. Mobile radiography services use small, lightweight, portable x-ray equipment with a digital detector [8]. The radiographer drives a vehicle equipped with a wheelchair ramp carrying the equipment and performs the examination with assistance from the nursing home staff in the resident’s room [8]. The images can be quality assessed on site and transferred to the radiology department for interpretation [8]. Image quality of examinations in nursing homes is adequate for making a diagnostic decision [10]. Mobile radiography services have been set up in a few countries, for instance in Australia, Canada, Norway, Sweden and the USA [8, 11–14]. However, further knowledge is needed about the outcomes of mobile radiography services for nursing homes residents.

The aim of this systematic review was to identify the outcomes of mobile radiography services for nursing home residents and for society in general.

## Methods

To the authors’ knowledge, this is the first systematic review aiming to identify outcomes of mobile radiography services for residents and society.

### Eligibility criteria

Empirical studies of mobile radiography services in a clinical setting for geriatric nursing home study populations were considered. The focus was on higher-level outcomes of diagnostic imaging on the levels “therapeutic”, “patient outcome” and “societal” efficacy, as described by Fryback and Thornbury [15]. In this review, the following designs were eligible: randomised controlled trials, non-randomised trials, descriptive studies, mixed-methods studies, socio-economic evaluations and qualitative studies.

### Literature search

The following databases were searched: MEDLINE Ovid, Cochrane Library, PubMed, Embase Ovid and Svemed+. The search strategy was developed in MEDLINE (Ovid) (Table 1), and was further adapted for the other databases. The terms used were derived from two categories: the population (nursing home resident) and intervention (mobile/portable radiography service OR mobile/portable x-ray service). The complete search strategy used is available in (Additional file 1: Table S1). The literature searches were carried out from December 2015 to February 2016, the last search on February 5^th^ 2016.

**Table 1:** Search strategy in MEDLINE (Ovid)

#	MEDLINE Ovid
1	nursing homes/or intermediate care facilities/or skilled nursing facilities/
2	Homes for the Aged/
3	(nursing adj (home* or facilit*)).tw.
4	(home? for the aged or home? for the elderly).tw.
5	((intermediate or long-term or longterm) adj care facilit*).tw.
6	2 or 3 or 4 or 5
7	exp Diagnostic Imaging/
8	((diagnostic or medical) adj (radio* or x-ray* or x ray*)).tw.
9	exp Radiography/
10	(mobile adj (radio* or x-ray* or x ray*)).tw.
11	(portable adj (radio* or x-ray* or x ray*)).tw.
12	exp Telemedicine/
13	(telemedicine adj (radio* or x-ray* or x ray*)).tw.
14	7 or 8 or 9 or 10 or 11 or 12 or 13
15	6 and 14

No language filters or date restrictions were used in the searches. The search was expanded by snowballing techniques screening for citations of the selected studies (Google scholar), reference lists and conference programmes. Grey literature like socio-economic evaluations were searched for using Google. The keywords used in Google are available in (Additional file 1: Table S2).

### Selection of records and methodological quality appraisal

The records were archived using Thomson Reuters EndNote X7.4 library and duplicates were removed. All titles and abstracts were screened by EK for eligibility, and a 10% sample was double-checked by KBL.

Mixed Methods Appraisal Tool (MMAT) was used for appraisal of the methodical quality of all studies, except economic evaluations. MMAT is considered appropriate for appraisal of qualitative, quantitative as well as mixed methods studies [16]. The Critical Appraisal Skills Program (CASP) tool [17] was used to appraise the methodological quality of the economic evaluation studies. EK and KBL read all the publications selected for full-text screening, appraised them, and agreed on the final grades and inclusion through discussions.

### Data extraction and synthesis

Data were extracted using a summary table based on recommendations by Støren [18]. The summary table was composed of the following categories: author, title and year, background, objective, research question, keywords, design, population, methods, results, conclusion, further questions, clinical implications and limitations. EK extracted data from all publications and KBL from 30% of the publications for quality assurance purposes.

A narrative synthesis was chosen due to the variety of methodologies used in the studies included in this review. This narrative synthesis included a familiarisation process of the results, methodological appraisal and transformation of quantitative data. Further, description and tabulation of data and performing a content analysis, and finally the authors discussed the synthesis through critical reflection until agreement was achieved [19].

## Results

Database searches, Google searches and snowballing identified 2548 individual records, which was reduced to 2238 after duplicates were removed. Two thousand two hundred twenty one excluded publications did not report on mobile radiography services. After screening, 17 full text publications were appraised.

**Fig. 1:**
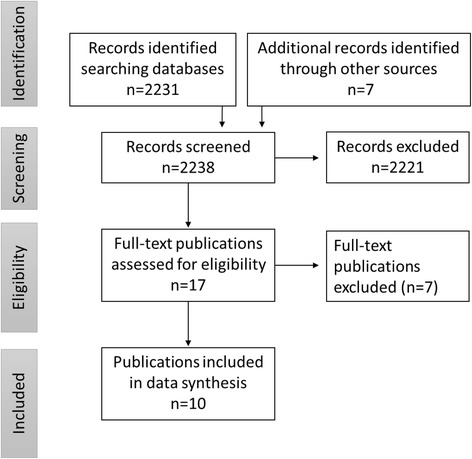
Flowchart of the selection process

Seven publications were excluded because of overlapping publications (from the same study), non-clinical settings, non-empirical designs or being technical or diagnostic accuracy efficacy assessments. An overview of excluded articles is available in Additional file 1: Table S3. No publications were excluded because of language. Figure 1 shows the selection process in detail.

Ten publications were included in this review: eight articles, one conference abstract, and one socio-economic evaluation.

### Characteristics of the included publications

Among the included publications are; one randomised controlled trial (RCT), one qualitative focus group interview study and three socio-economic analysis. The rest of the included publications reported on outcome of mobile radiography services based on different quantitative descriptive methods. Publications in English, Norwegian and German were included in the review. Further characteristics of the publications are listed in Table 2, along with scores for methodological quality. The detailed assessment of methodological quality, in MMAT or CASP forms, for each publication are available in (Additional file 1: Tables S4 and S5 respectively).

**Table 2:** Characteristics and methodological quality score of the publications included in the review

Author and year	Aim/objective	Design	Methods	Scope and type of data	Respondents	Area and nationality	MMAT/CASP grade^a^
Eklund 2011 [25]	Investigate the usefulness of a mobile radiography service for radiological assessment of patients in nursing homes from the patient and staff perspectives	Prospective, descriptive, quantitative study	• Questionnaire for nurses and residents • Registration form for image quality Telephone survey of outcome and treatment	123 nursing homes residents	Registered nurses at 25 nursing homes 62 residents	Lund, Sweden	****
Forat Sadry 2010 [22]	Investigate satisfaction with mobile services among referring physicians and nursing home staff	Prospective, descriptive, quantitative study	Questionnaire	318 nursing home residents using the mobile radiography service in 2007	Referring physicians and nursing home staff	BaselStadt, Baselland and Genf, Switzerland	*
Lærum 2005 [23]	Consequences for residents transferred to hospital for examination and treatment	Prospective, descriptive assessment	Questionnaire	714 nursing home residents	Nursing home staff at six nursing homes	Oslo, Norway	****
Lærum, Sager, Oswold 2005 [20]	Investigate feasibility of mobile services for residents, referring physicians and the nursing homes compared to outpatient services	Prospective, descriptive, quantitative study	Questionnaire	197 nursing home residents	Nursing home staff at 31 nursing homes	Oslo, Norway	***
Montalto 2015 [21]	Measure the impact of the mobile x-ray service on emergency department attendances by residents of residential aged care facilities who require plain X-ray services	Retrospective before-and-after cohort	Registry data analysis	Residents of 30 nursing homes frequently using the mobile x-ray service	n/a	Melbourne, Australia	****
Richauda 2011 [27]	Explore the quality of imaging and clinical outcomes of using mobile, light-weight x-ray equipment to provide radiologic examinations to frail elderly patients at home	Randomized controlled trail (RCT)	a) Confusion Assessment Method b) Delirium Rating Scale European Guidelines on Quality Criteria	69 immobilized or chair bound patients, acutely ill at intermediate or high risk of delirium in need of a radiological examination	7 radiologists	Torino, Italy	****
Thingnes & Stalsberg 2010 [26]	Explore aspects that nurses, nurse assistants and radiographers perceive important when implementing mobile radiography services to nursing homes	Qualitative	Focus group interviews	Health care personnel from one nursing home and one hospital	Radiographers, nurses and nurse assistants	Norway	***
Dozet 2015 [29] (abstract)	The aim of this study was to investigate whether mobile radiography was more cost-effective from a societal perspective, compared to hospital based radiological examinations.	Cost-effectiveness analysis	Prospective cost-minimization analysis	X-ray examinations in nursing homes (315 residents) compared to outpatient examinations (77 residents)	n/a	Lund, Sweden	*
Price Waterhouse Coopers 2006 [28]	Socio-economic cost-benefit analysis of shifting to mobile radiological services	Socio-economic cost-benefit evaluation	Literature review, interviews and valuing monetized effects	Registry data, reports and pilot project	Key personnel	Seven cities or areas of Norway	****
Randers 2005 [24]	Estimate socio-economic costs comparing two different ways of performing x-ray examinations of nursing home residents	Socio-economic cost evaluation	Costs analysis	Resources used and related cost statistics for mobile and stationary services	n/a	Norway	***

^a^In MMAT, papers are graded from 25% (one criteria met = *) to 100% (all criteria met = ****) [16]. In the CASP economic evaluation checklist, section B “How were costs and consequences assessed and compared?” publications were graded from 25% (1–2 criteria met = *) to 100% (all criteria met = ****)

**Fig. 2:**
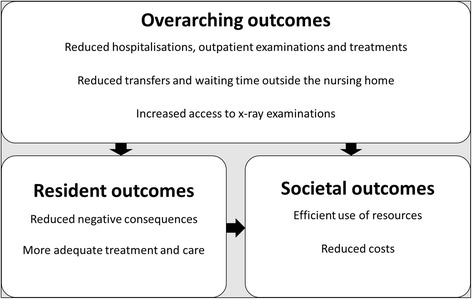
Outcomes of mobile radiography services. The arrows indicate links between overarching, residents’ and societal outcomes

In the analysis, it was found that the outcomes were on different levels and highly interlinked. Some outcomes were overarching in the sense that they are likely to influence or can explain other outcomes. Overarching outcomes of mobile radiography services are presented separately. Figure 2 shows an overview of the main findings and indicates how they may be interrelated.

### Overarching outcomes of mobile radiography services

First, mobile radiography services reduced the amount of hospitalisations, outpatients treatment and examinations in hospital or ER. Examination in the nursing home facilitated for instance, treatment of pneumonia in the nursing home instead of at the hospital or ER [5, 11, 20, 21]. Laerum and Sager [20] reported a 6% reduction in hospitalisation of nursing home residents after introducing mobile radiography services in Oslo. According to Montalto, Shay [21], nursing home residents’ presentation at the ER decreased by 11.5% the first year after introducing mobile radiography services in Melbourne.

Second, mobile radiography services reduced the use of ambulance and taxi transportation of nursing home residents for treatment or examination in hospitals or ERs. Two studies reported a 90–94% reduction in transfer of residents for outpatient x-ray examinations after introduction of mobile services [20, 22]. If the mobile radiography services had not been available, 50–88% of residents would have needed ambulance transportation and the rest would have needed a wheelchair taxi, regular taxi or private car [20, 22, 23].

One study reported on staff needed to accompany residents in transfer, and found that 75% of the residents needed nursing home staff to accompany them in transfer and while waiting [23]. Further, the next of kin accompanied 25% of the residents, and a few residents were accompanied by both staff and next of kin [23]. In addition, mobile radiography services reduced the time spent per examination. Randers [24] estimated a total of approximately 25 min per examination in a nursing home (from the arrival of the vehicle to departure). Two studies reported residents to be away for 4–5 h on average when going to an outpatient clinic in an urban area [23, 25]. According to Eklund, Klefsgård [25] and Thingnes and Stalsberg [26] most of this time was spent waiting or in transfer.

Finally, the number of necessary examinations performed increased when mobile radiography services were introduced. Laerum, Sager [20] reported 10% of the residents in their study were not able to be transferred for an outpatient examination. Further, residents often refuse to be transferred to hospital because they are scared according to Thingnes and Stalsberg [26]. Thus, mobile radiography services provided access to a radiological service for these residents and increased the number of residents receiving diagnostic services.

### Outcome for nursing home residents

There were two main outcomes for nursing home residents: First, avoiding hospitalisation, outpatient examination/treatment and transfer reduced the negative consequences for nursing home residents [23, 26, 27]. Second, radiological tests facilitated more adequate treatment and care [20, 25, 27].

Three studies reported the negative potential consequences for residents. According to Laerum, Åmdal [23] outpatient examinations were responsible for exhaustion and in certain cases confounded with confusion in 45% of the residents in their study [23]. Ricauda, Tibaldi [27] found that 17% of residents examined at the hospital developed delirium within a few hours after the examination. X-ray examination at the nursing home had an insignificant impact on residents [23] and none developed delirium [27]. In the qualitative study, the nurses and nurse assistants described residents to be confused, scared, restless and in pain when examined at the hospital. Furthermore, nursing home residents can cause disturbance for other patients at the radiology department [26].

Laerum, Åmdal [23] reported that the negative consequences for residents increased with the amount of time spent away from the nursing home. More than two and a half hours gave a significant (*p* < 0.001) increase in negative consequences for residents [23]. As previously described, residents are on average 4–5 h at the hospital for an outpatient x-ray examination [23, 25].

X-ray examinations provided important information for the treatment and care of nursing home residents. Three studies described the therapeutic outcome of examinations. For 58–70% of the examinations, the assumed diagnosis was confirmed and for 40% the tentative diagnosis was disproven [20, 27]. This was similar to examinations performed in a hospital [27]. According to Eklund, Klefsgård [25], 29% of the examinations in their study demonstrated significant pathology. Laerum, Sager [20] described that the findings of the mobile examinations had consequences for the medical treatment for 85% of the residents, and for care plans for 71% of the residents [20]. Hence, mobile services improved the adequacy of the treatment and care of nursing home residents.

### Societal outcomes

To invest in a vehicle and new equipment in addition to reorganising the way the radiographers work may have led to an increase in costs [24, 28]. However, the reduction in hospitalisations, transfers, staff accompanying residents and hospital/ER treatment reduced costs in both hospitals and nursing homes, thus for society as a whole [24, 28, 29].

When up to 75% of residents needed to be accompanied by healthcare staff and they were away on average 4–5 h [23, 25] the absence of staff have negative potential consequences for the other residents at the nursing home and the remaining nursing home staff, because the home is left short-handed [23, 26]. However, additional staff may be called in, which led to an additional increase in costs [28]. For society, 25% of the residents needed their next of kin to accompany them; this may have reduced effectiveness in the rest of society when employees have to take the day off work to take care of their family member [23].

Three publications from local projects in Norway and Sweden compared the cost of mobile radiography services with the cost of outpatient examinations, resulting in 30–60% cost reduction per examination. The size of the reduction depended on the distance between the nursing home and the hospital, in addition to the number of residents examined per visit [24, 28, 29].

## Discussion

The purpose of this systematic review was to create a better understanding of the outcomes of mobile radiography services compared to conventional x-ray examinations. Ten publications were included.

### Outcome of mobile radiography services

This review indicates three overarching outcomes of the introduction of mobile radiography services: a reduction in transfers from nursing homes to hospital or the ER for examination, treatment or care, a reduction in burdensome waiting time in hospital, and increased access to radiological procedures.

These overarching outcomes reduced the negative potential consequences for nursing home residents in need of x-ray examinations, and improved access to radiological tests for residents who for various reasons were unable to be transferred [20]. Furthermore, an x-ray examination facilitated more appropriate treatment and care [20, 24, 27]. This was of course dependent on the image quality being adequate for diagnosing. Studies comparing image quality in examinations carried out in hospitals and in nursing homes reported adequate diagnostic quality regardless of where the examination took place [10, 25, 27]. When examined at the nursing home, more residents would also be treated locally [20, 21]. This may have led to greater responsibilities for the nursing home staff, which may influence decisions about whether to send resident for acute treatment or examination at a hospital or to wait for the radiography services [26]. Conversely, treatment given locally facilitated coordination and continuity of treatment and care, which is important for this fragile population [23, 30].

For society, this review indicates that mobile radiography services could reduce healthcare costs by using resources more efficiently [24, 28, 29]. Both reduction in transfers with accompanying staff and changes in where treatment were given contributed to a cost reduction of 30–60% per examination [21, 24, 26, 28, 29]. Family members who accompanied residents to hospital, may be absent from work for one whole day. This would generate negative economic impact at a societal level.

Population demographics in the western world are changing with increasing life expectancy and fewer births. Ageing populations with increased healthcare needs and thus, increase in costs coupled with constrained resources creates efficiency pressures on healthcare services [31–33]. The European Commission calls for the use of telemedicine, new technology and a personalised healthcare system to meet these challenges [8, 33]. This review suggests that mobile radiography services can provide an effective alternative to outpatient x-ray examination for nursing home residents [24, 28, 29], in addition this can contribute to meet the challenges for healthcare efficiency. To date, only a few countries have introduced mobile radiography services. Barriers within the healthcare systems may prevent the establishment of these kind of services. Generally, in Europe telemedicine services are limited to local small-scale projects [9]. Hence, these barriers may be common for services that are organised differently than “ordinary” healthcare services. The way telemedicine services are organised may not fit the system of reimbursement from the health authorities. This may cause co-payment to be applied, which in turn may affect service provision or use [9]. Another reason may be lack of knowledge among decision-makers working in healthcare of the beneficial outcomes of mobile radiography services.

### Strengths and limitations

The search in the databases was systematic, and no language or date restrictions were used, thus the search strategy was exhaustive and it is likely to have been complete. The term mobile radiography services is also used to describe mobile radiography services within a hospital intensive care unit or at the emergency department. However, this did not cause any irrelevant hits because mobile/portable radiography/x-ray was combined with various terms for nursing home/home for the aged etc. Still, there were few studies and evidence was scarce. The variety in quality of the included publications limited the strength of the conclusions made in this review. The quality of evidence in systematic reviews are reflected in the level of confidence in the findings in the included studies [34]. The included publications were mostly related to programmes for introducing mobile radiography services in a community, which may have led to a bias towards positive outcomes of these services. However, with limited studies published it is important to identify existing knowledge in order to facilitate further research. Thus, publications were included despite suboptimal quality grading. Further, the types of studies included makes a narrative synthesis of results to be the best solution.

Notwithstanding its limitations, this review identified important benefits for nursing home residents and for society. Healthcare policies call for changes in organisation and efficiency, in addition to the use of new technology and telemedicine to reduce the strain on specialist healthcare [1, 9, 33, 35, 36]. Further research is needed to evaluate the outcome of these services in larger scale studies from different geographical areas (urban and rural). In addition, the outcome for individual residents and next of kin should be studied in more depth. The latter is presently unknown. There is a need for robust cost-effectiveness analyses from larger areas and more countries. Further, research is also needed to examine potential barriers to the implementation of telemedicine services in healthcare systems [9].

## Conclusion

This review indicates that mobile radiography services for nursing home residents in the western world are of comparable quality to hospital-based examinations and have clear potential benefits. Mobile radiography reduced transfers to and from hospital, increased the number of examinations carried out and facilitated timely diagnosis and access to treatments. Reduction in transfers, waiting times and exposure to unfamiliar environments contributed to the psychosocial well-being of nursing home residents and reduced disruption for carers and families of residents. Further research is needed to formally evaluate potential improvements in care quality and cost-effectiveness.

## Additional file

Additional file 1: Includes a complete search strategy, reasons for excluded articles and the MMAT and CASP checklists for all included articles. **Table S1.** Search strategy. **Table S2.** Searches in Google and Google Scholar. **Table S3.** Excluded articles with reasons. **Table S4.** Mixed Methods Appraisal Tool (MMAT) assessment of included studies. **Table S5.** CASP appraisal of economic evaluations included in the review. (DOCX 60 kb)

## Abbreviations

CASP: Critical Appraisal Skills Program
ER: Emergency room
MMAT: Mixed Methods appraisal tool
RCT: Randomized controlled trial
