# Supplementary material for: Mobile radiography services in nursing homes: a systematic review of residents’ and societal outcomes
Source: BMC Health Serv Res. 2017 Mar 23;17:231. doi: 10.1186/s12913-017-2173-8 (PMC5364720; doi:10.1186/s12913-017-2173-8)
Supplement: Additional file 1: — Includes a complete search strategy, reasons for excluded articles and the MMAT and CASP checklists for all included articles. Table S1. Search strategy. Table S2. Searches in Google and Google Scholar. Table S3. Excluded articles with reasons. Table S4. Mixed Methods Appraisal Tool (MMAT) assessment of included studies. Table S5. CASP appraisal of economic evaluations included in the review. (DOCX 60 kb) [file 12913_2017_2173_MOESM1_ESM.docx]

**Contents - Additional file**

[Table S1: Search strategy](#_Toc450286386)

[Table S2: Searches in Google and Google Scholar](#_Toc450286387)

[Table S3: Excluded articles with reasons](#_Toc450286388)

[Table S4: Mixed Methods Appraisal Tool (MMAT) assessment of included studies.](#_Toc450286389)

[Table S5: CASP appraisal of economic evaluations included in the review.](#_Toc450286390)

# Table S1: Search strategy

|  | | Cochrane Library 18.01.2016 | |  | |  |  |
| --- | --- | --- | --- | --- | --- | --- | --- |
| **ID** | | **Search** | | **Hits** | |  |  |
| #1 | | MeSH descriptor: [Nursing Homes] explode all trees | | 1067 | |  |  |
| #2 | | MeSH descriptor: [Homes for the Aged] explode all trees | | 505 | |  |  |
| #3 | | (nursing next (home* or facilit*)):ti,ab,kw | | 2327 | |  |  |
| #4 | | ("home? for the aged" or "home? for the elderly"):ti,ab,kw | | 545 | |  |  |
| #5 | | ((Intermediate or long-term or longterm) next care facilit*):ti,ab,kw | | 482 | |  |  |
| #6 | | #1 or #2 or #3 or #4 or #5 | | 2729 | |  |  |
| #7 | | MeSH descriptor: [Diagnostic Imaging] explode all trees | | 32141 | |  |  |
| #8 | | ((diagnostic or medical) next (radio* or x-ray* or x ray*)):ti,ab,kw | | 55 | |  |  |
| #9 | | MeSH descriptor: [Radiography] explode all trees | | 14110 | |  |  |
| #10 | | (mobile next (radio* or x-ray* or x ray*)):ti,ab,kw | | 11 | |  |  |
| #11 | | (portable next (radio* or x-ray* or x ray*)):ti,ab,kw | | 3 | |  |  |
| #12 | | MeSH descriptor: [Telemedicine] explode all trees | | 1374 | |  |  |
| #13 | | (telemedicine next (radio* or x-ray* or x ray*)):ti,ab,kw | | 1 | |  |  |
| #14 | | #7 or #8 or #9 or #10 or #11 or #12 or #13 | | 33514 | |  |  |
| #15 | | #6 and #14 | | 19 | |  |  |
| \| # \| MEDLINE Ovid 18.01.2016 \|  \| \| --- \| --- \| --- \| \| 1 \| nursing homes/ or intermediate care facilities/ or skilled nursing facilities/ \| 33459 \| \| 2 \| Homes for the Aged/ \| 11704 \| \| 3 \| (nursing adj (home* or facilit*)).tw. \| 25441 \| \| 4 \| (home? for the aged or home? for the elderly).tw. \| 2343 \| \| 5 \| ((intermediate or long-term or longterm) adj care facilit*).tw. \| 4314 \| \| 6 \| 2 or 3 or 4 or 5 \| 36646 \| \| 7 \| exp Diagnostic Imaging/ \| 1844124 \| \| 8 \| ((diagnostic or medical) adj (radio* or x-ray* or x ray*)).tw. \| 4712 \| \| 9 \| exp Radiography/ \| 673575 \| \| 10 \| (mobile adj (radio* or x-ray* or x ray*)).tw. \| 217 \| \| 11 \| (portable adj (radio* or x-ray* or x ray*)).tw. \| 356 \| \| 12 \| exp Telemedicine/ \| 17681 \| \| 13 \| (telemedicine adj (radio* or x-ray* or x ray*)).tw. \| 2 \| \| 14 \| 7 or 8 or 9 or 10 or 11 or 12 or 13 \| 1862541 \| \| 15 \| 6 and 14 \| 325 \| \| # \| Embase 1974 to 2016 Week 05 Ovid 05.02.2016 \|  \| | | | | | |  |  |
| 1 | nursing homes/ or intermediate care facilities/ or skilled nursing facilities/ | | 44749 | |  |  |  |
| 2 | Homes for the Aged/ | | 10530 | |  |  |  |
| 3 | (nursing adj (home* or facilit*)).tw. | | 32723 | |  |  |  |
| 4 | (home? for the aged or home? for the elderly).tw. | | 2990 | |  |  |  |
| 5 | ((intermediate or long-term or longterm) adj care facilit*).tw. | | 5518 | |  |  |  |
| 6 | 2 or 3 or 4 or 5 | | 46778 | |  |  |  |
| 7 | exp Diagnostic Imaging/ | | 126840 | |  |  |  |
| 8 | ((diagnostic or medical) adj (radio* or x-ray* or x ray*)).tw. | | 6246 | |  |  |  |
| 9 | exp Radiography/ | | 1007610 | |  |  |  |
| 10 | (mobile adj (radio* or x-ray* or x ray*)).tw. | | 265 | |  |  |  |
| 11 | (portable adj (radio* or x-ray* or x ray*)).tw. | | 395 | |  |  |  |
| 12 | exp Telemedicine/ | | 23252 | |  |  |  |
| 13 | (telemedicine adj (radio* or x-ray* or x ray*)).tw. | | 1 | |  |  |  |
| 14 | 7 or 8 or 9 or 10 or 11 or 12 or 13 | | 1128654 | |  |  |  |
| 15 | 6 and 14 | | 576 | |  |  |  |

|  | Svemed+ 18.01.2016 |  |
| --- | --- | --- |
| **#** | **Search** | **Hits** |
| 1 | exp:"nursing homes" | 1121 |
| 2 | care facility | 733 |
| 3 | home of the aged | 530 |
| 4 | home of the elderly | 470 |
| 5 | "long term care facility" OR "long-term care facility" | 4 |
| 6 | "intermediate care facility" | 8 |
| 7 | %231 OR %232 OR %233 OR %234 OR %235 | 2051 |
| 9 | exp:"Diagnostic imaging" | 4907 |
| 13 | Portable AND (radio* OR x-ray OR x ray) | 1 |
| 14 | Mobile AND (radio* OR x-ray OR x ray) | 4 |
| 15 | exp:"Telemedicine" | 321 |
| 16 | Telemedicine AND (radio* OR x-ray OR x ray) | 3 |
| 17 | (Diagnostic OR meidcal) AND (radio* OR x-ray OR x ray) | 113 |
| 19 | exp:"Radiography" | 2585 |
| 20 | %239 OR %2313 OR %2314 OR %2315 OR %2316 OR %2317 OR %2319 | 5213 |
| 21 | %237 AND %2320 | 29 |
|  | PubMed 05.02.2016 |  |
| Search | Query | Items found |
| #11 | Search (#5) AND #10 | 1592 |
| #10 | Search (((#6) OR #7) OR #8) OR #9 | 1150432 |
| #9 | Search (((telemedicine) AND (radio* or x-ray* or x ray*))) | 530 |
| #8 | Search (((portable) AND (radio* OR x-ray* OR "x ray"))) | 1360 |
| #7 | Search (((mobile) AND (radio* OR x-ray* OR """"x ray""""))) | 4695 |
| #6 | Search ((((diagnostic OR medical)) AND (imaging OR radio* OR x-ray* OR "x ray"))) | 1147199 |
| #5 | Search (((#1) OR #2) OR #3) OR #4 | 113775 |
| #4 | Search ("longterm care facility" OR "longterm care facilities" OR "long-term care facility" OR "long-term care facilities") | 4229 |
| #3 | Search ("intermediate care facility" OR "intermediate care facilities") | 782 |
| #2 | Search ("Home for the aged" OR "homes for the aged" OR "home for the elderly" OR "homes for the elderly") | 94187 |
| #1 | Search ("nursing home" OR "nursing homes" OR "nursing facility" OR "nursing facilities") | 43466 |

# Table S2: Searches in Google and Google Scholar

| 20.01.2016 - Google |  |
| --- | --- |
| Mobile røntgentjenester til sykehjemspasienter (Norwegian) [Mobile radiography services for nursing home patients] | 143 |
| "Mobile radiology services" "nursing homes" | 342 |
| "mobile x-ray services" "nursing homes" | 1250 |
| 22.02.16 – Google Scholar |  |
| Snowballing the eligible studies | 7 new |
| Total of records included in screening | 7 |

# Table S3: Excluded articles with reasons

| **Reference** | **Cause of exclusion** |
| --- | --- |
| Hellund, J.C., R. Tariq, and S. Sesseng, *Preliminary evaluation of the quality of the mobile radiographic images.* Michael quarterly, 2005. **2**(2): p.144-50 | Technical and/or diagnostic accuracy efficacy assessments |
| Loeb, M.B., et al., *Interobserver Reliability of Radiologists’ Interpretations of Mobile Chest Radiographs for Nursing Home–Acquired Pneumonia.* Journal of the American Medical Directors Association, 2006. **7**(7): p. 416-419. |  |
| CADTH, Mobile x-ray imaging versus fixed x-ray imaging in long-term care: clinical and cost-effectiveness. 2014. 4 Canadian Agency for Drugs and Technologies in Health (CADTH) http://onlinelibrary.wiley.com/o/cochrane/clhta/articles/HTA-32014000782/frame.html Accessed 18 Jan 2016 | Non-empirical |
| Lærum, F., *Sykehjemsrøntgen på hjul.* *[Nursing home radiography on wheels].* Michael quarterly, 2005. **2**: p. 168-89. |  |
| Minniti, D. et al., *Progetto di radiologia domiciliare della Regione Piemonte: 3 anni di sperimentazione. [R@dhome of Piedmont Region: three years of experimentation]* Ann Ig, 2013. **(Suppl. 1)**: p. 271-276. | Covered by already included articles |
| Lærum, F., et al., *Moving equipment, not patients: Mobile, net-based digital radiography to nursing home patients*. International Congress Series, 2005. **1281**: p. 922-925. |  |
| Verma, G., A.W. Chuck, and P. Jacobs, Tuberculosis screening for long-term care: a cost-effectiveness analysis. Int J Tuberc Lung Dis, 2013. **17**(9): p. 1170-7 | A screening program. |

# Table S4: Mixed Methods Appraisal Tool (MMAT) assessment of included studies.

| **Eklund et al** | MMAT criteria & one-page template | | | | |
| --- | --- | --- | --- | --- | --- |
| Types of mixed methods study components or primary studies | Methodological quality criteria (see tutorial for definitions and examples) | Responses | | | |
|  |  | Yes | No | Can’t tell | Comments |
| Screening questions | Are there clear qualitative and quantitative research questions (or objectives*), or a clear mixed methods question (or objective*)? | x |  |  | Aim |
|  | Do the collected data allow address the research question (objective)? E.g., consider whether the follow-up period is long enough for the outcome to occur (for longitudinal studies or study components). | x |  |  |  |
| 4. Quantitative descriptive | 4.1. Is the sampling strategy relevant to address the quantitative research question (quantitative aspect of the mixed methods question)? | x |  |  |  |
|  | 4.2. Is the sample representative of the population understudy? | x |  |  | All included |
|  | 4.3. Are measurements appropriate (clear origin, or validity known, or standard instrument)? | x |  |  |  |
|  | 4.4. Is there an acceptable response rate (60% or above)? | x |  |  | 100 % |
| Total score |  | 100% **** |  |  |  |

| **Lærum, Sager et al 2005** | MMAT criteria & one-page template | | | | |
| --- | --- | --- | --- | --- | --- |
| Types of mixed methods study components or primary studies | Methodological quality criteria (see tutorial for definitions and examples) | Responses | | | |
|  |  | Yes | No | Can’t tell | Comments |
| Screening questions | Are there clear qualitative and quantitative research questions (or objectives*), or a clear mixed methods question (or objective*)? | x |  |  | Aim |
|  | Do the collected data allow address the research question (objective)? E.g., consider whether the follow-up period is long enough for the outcome to occur (for longitudinal studies or study components). | x |  |  |  |
| 4. Quantitative descriptive | 4.1. Is the sampling strategy relevant to address the quantitative research question (quantitative aspect of the mixed methods question)? | x |  |  |  |
|  | 4.2. Is the sample representative of the population understudy? | x |  |  |  |
|  | 4.3. Are measurements appropriate (clear origin, or validity known, or standard instrument)? |  |  | x | Unclear |
|  | 4.4. Is there an acceptable response rate (60% or above)? | x |  |  |  |
| Total score |  | 75% *** |  |  |  |

| **Montalto et a**l 2015 | MMAT criteria & one-page template | | | | |
| --- | --- | --- | --- | --- | --- |
| Types of mixed methods study components or primary studies | Methodological quality criteria (see tutorial for definitions and examples) | Responses | | | |
|  |  | Yes | No | Can’t tell | Comments |
| Screening questions | Are there clear qualitative and quantitative research questions (or objectives*), or a clear mixed methods question (or objective*)? | x |  |  | Aim |
|  | Do the collected data allow address the research question (objective)? E.g., consider whether the follow-up period is long enough for the outcome to occur (for longitudinal studies or study components). | x |  |  |  |
| 4. Quantitative descriptive | 4.1. Is the sampling strategy relevant to address the quantitative research question (quantitative aspect of the mixed methods question)? | x |  |  |  |
|  | 4.2. Is the sample representative of the population understudy? | x |  |  | Top 30 users could overestimate the impact (risk of bias). |
|  | 4.3. Are measurements appropriate (clear origin, or validity known, or standard instrument)? | x |  |  |  |
|  | 4.4. Is there an acceptable response rate (60% or above)? | x |  |  |  |
| Total score |  | 100% **** |  |  |  |

| **Lærum, Åmdal et al 2005** | MMAT criteria & one-page template | | | | |
| --- | --- | --- | --- | --- | --- |
| Types of mixed methods study components or primary studies | Methodological quality criteria (see tutorial for definitions and examples) | Responses | | | |
|  |  | Yes | No | Can’t tell | Comments |
| Screening questions | Are there clear qualitative and quantitative research questions (or objectives*), or a clear mixed methods question (or objective*)? | x |  |  |  |
|  | Do the collected data allow address the research question (objective)? E.g., consider whether the follow-up period is long enough for the outcome to occur (for longitudinal studies or study components). | x |  |  | Limited data compared to the scope of the third research question. |
| 4. Quantitative descriptive | 4.1. Is the sampling strategy relevant to address the quantitative research question (quantitative aspect of the mixed methods question)? | x |  |  | Additional information provided from the corresponding author |
|  | 4.2. Is the sample representative of the population understudy? | x |  |  | Additional information provided from the corresponding author |
|  | 4.3. Are measurements appropriate (clear origin, or validity known, or standard instrument)? | x |  |  | Additional information provided from the corresponding author |
|  | 4.4. Is there an acceptable response rate (60% or above)? | x |  |  | Additional information provided from the corresponding author |
| Total score |  | 100%**** |  |  |  |

| **Ricauda et al, 2011** | MMAT criteria & one-page template (to be included in appraisal forms) | | | | |
| --- | --- | --- | --- | --- | --- |
| Types of mixed methods study components or primary studies | Methodological quality criteria (see tutorial for definitions and examples) | Responses | | | |
|  |  | Yes | No | Can’t tell | Comments |
| Screening questions | Are there clear qualitative and quantitative research questions (or objectives*), or a clear mixed methods question (or objective*)? | x |  |  | Aim |
|  | Do the collected data allow address the research question (objective)? E.g., consider whether the follow-up period is long enough for the outcome to occur (for longitudinal studies or study components). | x |  |  |  |
| 2. Quantitative randomized controlled (trials) | 2.1. Is there a clear description of the randomization (or an appropriate sequence generation)? | x |  |  |  |
|  | 2.2. Is there a clear description of the allocation concealment (or blinding when applicable)? | x |  | x | Blinded for assessment of image quality, but unclear for post examination Delirium Rating Scale assessment |
|  | 2.3. Are there complete outcome data (80% or above)? | x |  |  |  |
|  | 2.4. Is there low withdrawal/drop-out (below 20%)? | x |  |  |  |
| Total score |  | 100%**** |  |  |  |

| **Forat et al 2010** | MMAT criteria & one-page template | | | | |
| --- | --- | --- | --- | --- | --- |
| Types of mixed methods study components or primary studies | Methodological quality criteria (see tutorial for definitions and examples) | Responses | | | |
|  |  | Yes | No | Can’t tell | Comments |
|  |  |  |  |  |  |
| Screening questions | Are there clear qualitative and quantitative research questions (or objectives*), or a clear mixed methods question (or objective*)? | x |  |  | Aim |
|  | Do the collected data allow address the research question (objective)? E.g., consider whether the follow-up period is long enough for the outcome to occur (for longitudinal studies or study components). | x |  |  |  |
| 4. Quantitative descriptive | 4.1. Is the sampling strategy relevant to address the quantitative research question (quantitative aspect of the mixed methods question)? |  |  | x | Unclear connection between aim and questions asked |
|  | 4.2. Is the sample representative of the population understudy? | x |  |  |  |
|  | 4.3. Are measurements appropriate (clear origin, or validity known, or standard instrument)? |  | x |  |  |
|  | 4.4. Is there an acceptable response rate (60% or above)? |  | x |  |  |
| Total score |  | 25%* |  |  |  |

| **Thingnes & Stalsberg** 2010 | MMAT criteria & one-page template | | | | |
| --- | --- | --- | --- | --- | --- |
| Types of mixed methods study components or primary studies | Methodological quality criteria (see tutorial for definitions and examples) | Responses | | | |
|  |  | Yes | No | Can’t tell | Comments |
| Screening questions | Are there clear qualitative and quantitative research questions (or objectives*), or a clear mixed methods question (or objective*)? | x |  |  |  |
|  | Do the collected data allow address the research question (objective)? E.g., consider whether the follow-up period is long enough for the outcome to occur (for longitudinal studies or study components). | x |  |  |  |
| 1. Qualitative | 1.1. Are the sources of qualitative data (archives, documents, informants, observations) relevant to address the research question (objective)? | x |  |  |  |
|  | 1.2. Is the process for analyzing qualitative data relevant to address the research question (objective)? | x |  |  |  |
|  | 1.3. Is appropriate consideration given to how findings relate to the context, e.g., the setting, in which the data were collected? | x |  |  |  |
|  | 1.4. Is appropriate consideration given to how findings relate to researchers’ influence, e.g., through their interactions with participants? |  | x |  |  |
| Total score |  | 75% *** |  |  |  |

# Table S5: CASP appraisal of economic evaluations included in the review.

| **Price Waterhouse Coopers, 2006** |  | Yes | Can’t tell | No |
| --- | --- | --- | --- | --- |
| \| Is the economic evaluation valid? \| \| --- \| | 1. Was a well-defined question posed? | X |  |  |
|  | 1. Was a comprehensive description of the competing alternatives given? | X |  |  |
| \| How were costs and consequences assessed and compared? \| \| --- \|  \|  \| \| --- \| | 1. Does the paper provide evidence that the programme would be effective (i.e. would the programme do more good than harm? | X |  |  |
|  | 1. Were the effects of the intervention identified, measured and valued appropriately? | X |  |  |
|  | 1. Were all important and relevant resources required and health outcome costs for each alternative identified, measured in appropriate units and valued credibly? | X |  |  |
|  | 1. Were costs and consequences adjusted for different times at which they occurred (discounting)? | X |  |  |
|  | 1. What were the results of the evaluation? | X |  |  |
|  | 1. Was an incremental analysis of the consequences and cost of alternatives performed? | X |  |  |
|  | 1. Was an adequate sensitivity analysis performed? | X |  |  |
| Will the results help in purchasing services for local people? | 1. Is the programme likely to be equally effective in your context or setting? | X |  |  |
|  | 1. Are the costs translatable to your setting? | X |  |  |
|  | 1. Is it worth doing in your setting? | X |  |  |
| Total score |  | 100% | **** |  |

| **Randers 2005** |  | Yes | Can’t tell | No | Comment |
| --- | --- | --- | --- | --- | --- |
| \| Is the economic evaluation valid? \| \| --- \| | 1. Was a well-defined question posed? | X |  |  |  |
|  | 1. Was a comprehensive description of the competing alternatives given? | X |  |  |  |
| \| How were costs and consequences assessed and compared? \| \| --- \|  \|  \| \| --- \| | 1. Does the paper provide evidence that the programme would be effective (i.e. would the programme do more good than harm? | X |  |  | To the extent relevant in this paper |
|  | 1. Were the effects of the intervention identified, measured and valued appropriately? | X |  |  | To the extent relevant in this paper |
|  | 1. Were all important and relevant resources required and health outcome costs for each alternative identified, measured in appropriate units and valued credibly? | X |  |  | To the extent relevant in this paper |
|  | 1. Were costs and consequences adjusted for different times at which they occurred (discounting)? |  |  | X |  |
|  | 1. What were the results of the evaluation? | X |  |  |  |
|  | 1. Was an incremental analysis of the consequences and cost of alternatives performed? | X |  |  |  |
|  | 1. Was an adequate sensitivity analysis performed? |  |  | X |  |
| Will the results help in purchasing services for local people? | 1. Is the programme likely to be equally effective in your context or setting? | X |  |  |  |
|  | 1. Are the costs translatable to your setting? | X |  |  |  |
|  | 1. Is it worth doing in your setting? | X |  |  |  |
| Total score |  | 75% | *** |  |  |

| **Dozet, 2015 (abstract)** |  | Yes | Can’t tell | No | Comment |
| --- | --- | --- | --- | --- | --- |
| \| Is the economic evaluation valid? \| \| --- \| | 1. Was a well-defined question posed? | X |  |  |  |
|  | 1. Was a comprehensive description of the competing alternatives given? | X |  |  |  |
| \| How were costs and consequences assessed and compared? \| \| --- \|  \|  \| \| --- \| | 1. Does the paper provide evidence that the programme would be effective (i.e. would the programme do more good than harm? |  | X |  | Corresponding author has been contacted for more information – but this has not been given |
|  | 1. Were the effects of the intervention identified, measured and valued appropriately? |  | X |  |  |
|  | 1. Were all important and relevant resources required and health outcome costs for each alternative identified, measured in appropriate units and valued credibly? |  | X |  |  |
|  | 1. Were costs and consequences adjusted for different times at which they occurred (discounting)? |  | X |  |  |
|  | 1. What were the results of the evaluation? | X |  |  |  |
|  | 1. Was an incremental analysis of the consequences and cost of alternatives performed? | X |  |  |  |
|  | 1. Was an adequate sensitivity analysis performed? |  | X |  |  |
| Will the results help in purchasing services for local people? | 1. Is the programme likely to be equally effective in your context or setting? | X |  |  |  |
|  | 1. Are the costs translatable to your setting? | X |  |  |  |
|  | 1. Is it worth doing in your setting? | X |  |  |  |
| Total score |  | 25% | * |  |  |
